# Neonicotinoid Analysis in Sunflower (*Helianthus annuus*) Honey Samples Collected around Tekirdag in Turkey

**DOI:** 10.1155/2023/9429449

**Published:** 2023-03-16

**Authors:** Nurullah Ozdemir, Mustafa Necati Muz

**Affiliations:** ^1^Tekirdag Namık Kemal University, Veterinary Faculty, Department of Pharmacology and Toxicology, Tekirdag, Turkey; ^2^Tekirdag Namık Kemal University, Veterinary Faculty, Department of Parasitology, Tekirdag, Turkey

## Abstract

In recent years, the widespread use of neonicotinoids in agricultural areas has caused environmental pollution due to its lower toxicity to mammals. Honey bees, which are considered as biological indicators of environmental pollution, can carry these pollutants to the hives. Forager bees returning from sunflower crops that have been treated with neonicotinoids treated sunflower fields cause residue accumulation in the hives, which reason colony-level adverse effects. This study analyses neonicotinoid residues in sunflower (*Helianthus annuus*) honey sampled by beekeepers from Tekirdag province. Honey samples have been subjected to liquid-liquid extraction methods before liquid chromatography-mass spectrometry (LC-MS/MS). The method validation was carried out to fulfill all the necessary requirements of procedures SANCO/12571/2013. Accuracy was in the range of 93.63–108.56%, for recovery in the range of 63.04–103.19%, and for precision in the range 6.03–12.77%. Detection and quantification limits were determined according to the maximum residue limits of each analyte. No neonicotinoid residues were found above the maximum residue limit in the sunflower honey samples analysed.

## 1. Introduction

Neonicotinoids are insecticides that show nicotine-like stimulating effects by binding to nicotinic acetylcholine receptors in the CNS. Neonicotinoids are more toxic to insects because they bind more tightly and irreversibly to nicotinic acetylcholine receptors in insects than in mammals [1, 2].

Neonicotinoid-type plant protection products are widely used in the agricultural field, especially for preserving seeds due to their less toxic effect on mammals; they also have unintended consequences on nontarget organisms like agrarian pollinators [3]. Pollution is also detected in the fields where neonicotinoid-treated seeds are planted and in the application area's surface or public drinking water sources [4].

Commercial beekeepers move millions of honeybee colonies to sunflower fields to get sunflower honey during the blooming sessions. During these visits, honey bees carry possible pesticide residues in the environment to the hives by means of nectar and pollen. Neonicotinoids can affect the bee's ability to fly for feeding purposes, such as learning and remembering ways to reach food sources. Therefore, it can be considered as part of the important role in colony collapse disorder [5].

The neonicotinoids have been restricted in the first Europe due to the adverse environmental effects resulting from a severe decrease in honey bees, insect populations, and bird species and numbers. The European Union established maximum residue limits (MRLs) for acetamiprid, clothianidin, imidacloprid, thiacloprid, and thiamethoxam in the range of 10–200 ng·k^−1^ [6].

The importance of sunflower honey in the global honey trade is different; its flavour is not strong dominantly. Therefore, it blends well with other honey types, and it is one of the most suitable and economic honey for commercial blending. Honey, which must comply with EU standard norms, must be reliable regarding food safety and public health. High pesticide concentrations can cause high mortality in bees, loss of colonies, and honey production unsuitable for food safety [7].

For public health, pesticides in honey and other food have become a severe health and safety checkpoint worldwide, and demands for detecting chemicals that may pose an environmental risk have increased in recent years. Successful results have been obtained in analysing multiple residues of antibiotics and pesticides in honey using liquid chromatography-mass spectrometry.

Different extraction methods and devices have been used to determine neonicotinoid residues in sunflower honey. Liquid-liquid extraction (LLE) [8], solid-phase extraction (SPE) [9, 10], QuEChERS extraction [11–13], and dispersive liquid-liquid microextraction (DLLME) [14] can be counted among the effective extraction methods used. A high-pressure liquid chromatography diode array detector (HPLC/DAD) [15], a gas chromatography-mass spectrometer (GC-MS/MS) [13], and liquid chromatography-mass spectrometry (LC-MS/MS) are used for neonicotinoid analyses [12, 16].

This study aims to determine neonicotinoid residues in sunflower honey collected from 33 different beekeepers around Tekirdag province. According to the results obtained, a risk assessment will be made in terms of public health. The study is the first to investigate neonicotinoid pesticide residues in honey around Tekirdag. It is thought that it will importantly contribute to databases in this regard.

## 2. Materials and Methods

### 2.1. Chemicals and Reagents

The following standards for neonicotinoids were used: imidacloprid (99.9%), acetamiprid (99.9%), clothianidin (99.9%), nitenpyram (99.9%), thiacloprid (99.9%), dinotefuran (98.8%), and thiamethoxam (99.6%). The purity of all compounds was greater than 98%. The internal standard of clothianidin-d3 (97%) was obtained from Sigma-Aldrich. Acetonitrile for HPLC was obtained from Fluka. Dichloromethane and glacial acetic acid for analysis were obtained from Merck.

### 2.2. Standards and Solutions

#### 2.2.1. Standard Stock Solution (1 mg·mL^−1^ Each)

10 mg of each reference standard was weighed into a 10 mL Class A graduated flask. Sufficient methanol was added up to the mark. Stock solutions were protected at −18°C.

#### 2.2.2. S_2_-Working Standard Solution (10 ng·mL^−1^ Each)

0.1 mL of each reference standard stock solution was taken and placed in a 10 mL measuring balloon. Sufficient methanol was added up to the mark. The prepared working solutions were stored at 4–6°C.

#### 2.2.3. S_4_-Standard Working Solution (100 ng·mL^−1^ Each)

0.1 mL of each S_2_-standard working solution was taken and placed in a 10 ml measuring flask. Sufficient methanol was added up to the mark. All standard working solutions were stored at 4–6°C. This prepared solution was used to define reference standards in the MS detector.

#### 2.2.4. Reference Standard Solution Mix for the Spike

Spiking solutions were prepared at 10 ng·mL^−1^ for clothianidin, dinotefuran, nitenpyram, and thiamethoxam, 50 ng·mL^−1^ for acetamiprid and imidacloprid, and 200 ng·mL^−1^ for thiacloprid, according to the levels of maximum residue limit (MRL) in the honey, respectively (Table 1). One mL of clothianidin, dinotefuran, nitenpyram, and thiamethoxam S_4_ working solution, 5 mL of acetamiprid and imidacloprid S_4_ working solution, and 0.2 mL of thiacloprid S_2_ working solution were put into a 10 mL measuring balloon and filled with methanol up to the mark.

#### 2.2.5. Internal Standard Solution

Clothianidin-d_3_ was used as an internal standard at a 10 ng·mL^−1^.

#### 2.2.6. Mobile Phase *A*

Acetonitrile was used as a mobile phase *A*.

#### 2.2.7. Mobile Phase *B*

2 mL of acetic acid were placed in a 1 L flask and the reagent diluted with water to the marked line. The mobile phases were degassed in an ultrasonic bath for 15 min.

### 2.3. Collection of Samples

During sunflower honey harvest, honey samples were collected from supers at each of the 33 stationary apiaries in July/August of 2015 from 10 different district centres of Tekirdag (40°58′41″N, 27°30′42″E). All samples were confirmed to be sunflower honey by pollen analysis.

### 2.4. Extraction of Honey Samples

The extraction method [14] was used for the honey samples. Briefly, two grams of each honey sample were weighed into 15 mL polypropylene centrifuge tubes and an internal standard solution (100 *μ*L) was added to the tubes. The mixed standard spiking solutions were added (50, 100, 150, and 200 *μ*L) to control the quality of the samples. 0.5 mL of acetonitrile and 2.0 mL of dichloromethane were placed in each tube. The tubes were mixed by vortex for 1 minute, incubated in an ultrasonic bath for 10 minutes, returned to the vortex for 1 minute, and centrifuged at 2,500g for 5 minutes, 6 mL of supernatant was then removed using a pipette and transferred into glass tubes. The organic fraction was evaporated to dryness in a stream of nitrogen at 40°C within a water bath. Two mL of mobile phase was added onto the dry residue and mixed by vortex for two minutes. The result was filtered into an autosampler vial using a 0.2 *µ*m syringe filter.

### 2.5. Instrumentation

Analyses were performed on AB Sciex 3200 QTRAP brand/model high-pressure liquid chromatography-mass spectrometry equipment controlled by Analyst 1.6.1 software. An Agilent Poroshell 120 SB: C18 2.7 *µ*m 100 × 3.0 mm column was used for chromatographic separation. Acetonitrile (A) and water acidified with 0.2% acetic acid were used as the mobile phase. The linear gradient mobile phase; 0-1 min 80% *A*, 1–3.3 min 50% *A*, and 3.4–6 min 80% *A*, and flow rate of 0.3 mL/min. The injection volume was 10 *µ*L, and the column temperature was 40°C.

### 2.6. Mass Spectrometry

The MS/MS detector parameters and precursor-product ions of each referenced standard substance are shown in Table 2. A capillary voltage of 5500 V, nebulizer gas of 7 psi, curtain gas of 30 psi, heater gas of 50 psi, and collision gas of 50 psi were set. The temperature of the TurboIonSprey module was set at 400°C.

Ionization was performed in positive ion mode using the electrospray ionization (ESI) module.

## 3. Results and Discussion

### 3.1. Method Validation

The selectivity/sensitivity, linearity, limit of detection (LOD) and limit of quantitation (LOQ), decision limit (CC*α*), detection capability (CC*β*), accuracy, and recovery parameters were calculated for the method validation.

#### 3.1.1. Specificity/Selectivity

**Figure 1:**
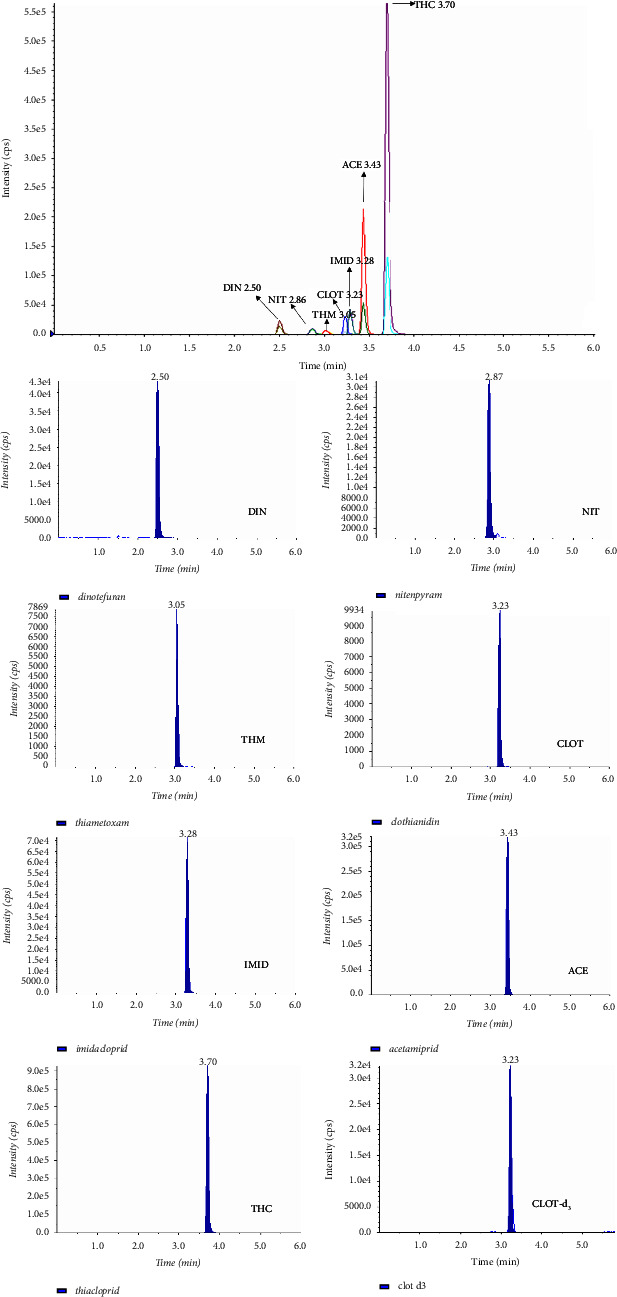
Chromatograms of total ion chromatogram and fortified spiked honey samples (according to MRL or LOQ). DIN, dinotefuran; NIT, nitenpyram; THM, thiamethoxam; CLOT, clothianidin; IMID, imidacloprid; ACE, acetamiprid; THC, thiacloprid; CLOT-d_3_, clothianidin-d_3_.

Blank samples were analysed by loading different standard substances; no interference was observed in the retention times. It was concluded that the analysis method was suitable for selectivity/sensitivity. The chromatogram obtained from loading at the MRL level is shown in Figure 1.

#### 3.1.2. Linearity

To determine the linearity of the method, six parallel analyses were performed using four different concentration points at 0.5, 1, 1.5, and 2 MRL levels in accordance with the MRL level in honey. Calibration curves for each standard substance were created. The *r*^2^ value in the calibration curve of each standard item was found to be between 0.9908 and 0.9984 (Table 1).

#### 3.1.3. Limit of Detection (LOD) and Limit of Quantification (LOQ)

To determine the limit of detection and the limit of quantification, 10 parallel analyses were performed at 0.5 MRL. The results obtained are shown in Table 1.

#### 3.1.4. Decision Limit (CC_*α*_) and Detection Capability (CC_*β*_)

The decision limit (CC_*α*_) and detection capability (CC_*β*_) were calculated using the results obtained from the study linearity and are shown in Table 1.

#### 3.1.5. Accuracy

The accuracy was calculated using the study linearity and recovery results shown in Table 3.

#### 3.1.6. Recovery

To determine recovery, analysis was performed according to the blank fortified sample at levels 0.5, 1, 1.5, and 2 MRL shown in Table 3.

According to the sunflower honey samples analysis, the MRL value was not detected for any neonicotinoid residue. The data under the maximum residue levels have not been evaluated. The results of the analysis of the honey samples are shown in Table 4.

### 3.2. Discussion

The analytical method was validated in conformity to the SANCO 12571/2013, results are presented in Tables 1 and 3. The matrix-matched curves showed good linearity (*r*^2^ > 0.99) for all the analytes. The concentrations of the analytes were obtained directly from the matrix calibration curve with the use of internal standards. The selectivity of the method was found to be gratifying with no interference peaks from endogenous compounds in the retention time of the target analytes in honey samples. Precision, expressed as the repeatability, gave the RSD values in agreement with the SANCO criteria of RSD ≤20%. The RSD, were in the range of 6.3–12.77% for honey samples. Satisfactory average recoveries were calculated used of the internal standards. The average recovery result ranged 63.04–103.19% for honey samples, and is in accordance with the SANCO validation guideline of recovery, which should be in the range of 60–140%.

The results of analysis of honey samples: no neonicotinoid pesticide residues were detected above the maximum residue limits in honey samples collected from Tekirdag province and its surroundings. Among the possible reasons, a neonicotinoid pesticide type drug is not used in and around Tekirdag. This may have been caused by agricultural producers' avoidance of the use of neonicotinoid pesticides, as some countries in Europe have banned or restricted the use of neonicotinoid pesticides.

Previous studies published about the confirmation method and validation of the residues of neonicotinoids in honey are summarised below.

In a study by Kavanagh et al. in Irish honey samples, imidacloprid was found to be the most common neonicotinoid (found in 13.43% of honey samples), followed by clothianidin (12.40%) and thiacloprid (11.37%). They concluded that the frequency of imidacloprid in honey samples may not be limited to its use in the agricultural field but may also occur due to its presence in a range of commercial products used in sports and recreational lawn care products, herb care homes, home gardens, and locally public parks [17].

In Austria, acetonitrile extraction and dispersive solid-phase extraction (QuEChERS type) were used in Tanner and Czerwenka's analytical method to detect neonicotinoid residues in honey. Residues of acetamiprid, thiacloprid, and thiamethoxam were detected in Austrian honey samples; however, no sample exceeded the maximum residue limits. Flower honey samples contained more neonicotinoid residues than forest honey samples [18]. It is seen that the level of neonicotinoid pesticide residues in honeys of Austria detected in this study are below the maximum residue limits in line with the results obtained from our study.

Ligor et al. developed a method using QuEChERS extraction and UHPLC/UV to determine neonicotinoid residues in honey samples. The method was applied to honey collected from Poland and other countries. 53 honey samples were analysed, and neonicotinoids were detected at concentrations higher than the LOQ in 19 honey samples from Australia (3 samples), Brazil (1 sample), Italy (1 sample), and Poland (12 samples). No neonicotinoid residues were detected in the Turkish honey sample [19]. The absence of neonicotinoid residues in the analysis of honey samples from Turkey seems to be in line with the result of our study.

In the study by Woodcock, we evaluated the effectiveness of this policy in reducing the risk of exposure to honeybees by collecting 130 honey samples from beekeepers in the UK before (2014: *N* = 21) and after (2015: *N* = 109) the enactment of the moratorium. Neonicotinoids were present in approximately half of the honey samples taken before the moratorium and in more than one-fifth of the honey samples taken after the moratorium. Clothianidin was the most frequently detected neonicotinoid [20].

A 3-year field study was conducted in France from 2002 to 2005 to examine pesticide residues found in colonies and honeybee (*Apis mellifera L*.) colony health by Chauzat et al. No pesticide residues were detected in 12.7% of the sampling periods. It was reported that no statistical relationship was found between colony mortality and pesticide residues. Imidacloprid residues were frequently detected in pollen, honey, and honeybee samples [21].

Mrzlikar et al. developed a reliable analytical method using two extraction techniques (SPE, QuEChERS) and LC-MS/MS (SRM) for five neonicotinoids in 51 honey samples collected between 2014 and 2016. Despite being banned in the country in 2011, residues of acetamiprid and thiacloprid were detected in low contamination [12].

An average of 8.2 ng·g^−1^ clothianidin and 17.2 ng·g^−1^ thiamethoxam were detected in 68% and 75% of honey samples, respectively, from hives located 30 km from Saskatchewan City in Canada. Moreover, clothianidin was found in >50% of bee and pollen samples. Imidacloprid was detected in ∼30% of honey samples [11].

In a study by Han et al., a total of 94 honey samples were selected from the Chinese market, based on the production region and sales volume in 2020. Neonicotinoids and their metabolites were detected in 97.9% of honey samples. Acetamiprid, thiamethoxam, and imidacloprid were the top three neonicotinoids in honey with detection frequencies of 92.6%, 90.4%, and 73.4%, respectively [22].

A study conducted in North America from 2007 to 2008 examined the effects of pesticides on the health of bee colonies. 1% of 208 wax samples, 17.7% of 350 pollen samples, and 0.0% of 140 honey samples were detected as having imidacloprid residues [23].

Residues of neonicotinoids were investigated in honey, pollen, and bee samples sampled in Greece between 2011 and 2013, while any residue did not detect in the honey samples. However, 0.7–14.7 ng·g^−1^ clothianidin in bee samples in 2011, 6.1–69.04 ng·g^−1^ in pollen samples, and 2.7–39.9 ng·g^−1^ was detected in 2012 bee samples and 308.3–1273 ng·g^−1^ clothianidin in pollen samples [24]. The absence of neonicotinoid residues in the analysis of honey samples from Turkey seems to be in line with the result of our study.

Residues of neonicotinoid products restricted in the European Union were not found in honey samples from Tekirdag on the European side of Turkey. According to the results of the study, we can say that the use of neonicotinoid products has decreased in our country.

## 4. Conclusions

According to the analysis results of 33 sunflower honey samples collected from around and Tekirdag province are free of any neonicotinoid residues exceeding the maximum residue limits were detected. It was understood that in further studies, more honey samples should be analysed as well as other hive products.

## Figures and Tables

**Table 1:** Summary of LOD, LOQ, CC*α*, and CC*β*.

Analyte	Calibration range (*μ*g kg^−1^)	Linearity (*r*^2^)	Limit of detection (LOD)	Limit of quantification (LOQ)	Decision limit (CC*α*)	Detection capability (CC*β*)	MRL (EU) (*μ*g kg^−1^)
Dinotefuran	5–20	0.9908	6.25	9.45	10.74	11.50	10
Nitenpyram	5–20	0.9984	6.15	9.77	11.08	12.17	10
Thiamethoxam	5–20	0.9963	6.42	9.72	11.48	12.94	10
Clothianidin	5–20	0.9910	5.42	8.70	10.94	11.88	10
Imidacloprid	25–100	0.9956	26.63	36.94	57.18	64.37	50
Acetamiprid	25–100	0.9954	29.18	43.20	56.86	63.73	50
Thiacloprid	100–400	0.9922	102.67	155.34	221.11	242.22	200

**Table 2:** MS/MS detector parameters and retention times (RT).

Analytes	RT	Precursor ion (*m*/z)	Product ion (*m*/z)	DP (volts)	EP (volts)	CEP (volts)	CE (volts)	CXP (volts)
Dinotefuran	2.4	203.08	129.1^*∗*^	36	8	18	15	4
			114.1	36	8	18	17	4

Nitenpyram	2.8	271.12	225.20^*∗*^	36	5.5	16	15	4
			56.00	36	5.5	16	49	4
Thiamethoxam	3.0	292.00	211.10^*∗*^	31	11.5	16	15	4
			132.10	31	11.5	16	25	4

Clothianidin	3.2	250.07	132.00^*∗*^	41	7.5	14	19	4
			169.10	41	7.5	14	15	4

Clothianidin-d3 (IS)	3.2	253.01	132.00	41	8	14	23	4

Imidacloprid	3.3	256.10	290.10^*∗*^	36	9	14	19	4
			175.10	36	9	14	21	4

Acetamiprid	3.4	223.07	126.20	41	9	12	27	4
			99.20	41	9	12	47	4

Thiacloprid	3.7	253.06	126.20	46	12	14	29	4
			99.10	46	12	14	53	4

^*∗*^Confirmative ion. DP: declustering potential, EP: entrance potential, CEP: cell exit potential, CE: collision energy, CXP: collision cell exit potential, IS: internal standard.

**Table 3:** Accuracy and recovery of neonicotinoids in honey samples.

	Added amount (*μ*g kg^−1^)	Mean amount calculated (*μ*g kg^−1^)	RSD (%)	Accuracy (%)	Recovery (%)
Dinotefuran	5	5.25	11.56	105.00	84.08
	10	10.86	7.11	108.56	82.57
	15	15.79	6.16	105.29	82.52
	20	18.72	6.92	93.63	78.94

Nitenpyram	5	5.13	7.98	102.75	87.92
	10	10.42	7.27	104.20	92.75
	15	15.71	12.77	104.80	74.75
	20	19.08	8.20	95.30	63.71

Thiamethoxam	5	4.98	8.27	99.69	71.36
	10	10.03	9.75	100.25	84.50
	15	15.25	7.27	101.59	78.63
	20	19.79	8.21	98.74	73.02

Clothianidin	5	5.06	11.94	101.34	63.04
	10	10.13	10.94	101.31	73.65
	15	15.18	10.09	101.23	70.72
	20	19.75	6.03	98.81	86.06

Imidacloprid	25	23.55	10.92	94.10	74.68
	50	51.59	6.48	103.21	74.98
	75	77.64	7.26	103.63	77.57
	100	96.70	6.61	96.70	71.26

Acetamiprid	25	23.61	10.34	94.50	103.19
	50	52.36	8.42	104.70	95.83
	75	79.23	9.37	105.53	94.94
	100	94.70	8.56	94.70	93.64

Thiacloprid	100	100.23	7.13	100.23	94.12
	200	209.00	9.30	104.45	92.00
	300	318.00	10.60	105.99	92.21
	400	377.88	10.84	94.51	90.56

**Table 4:** The results of analysis honey samples.

No.	Dinotefuran (ug·kg^−1^)	Nitenpyram (ug·kg^−1^)	Thiamethoxam (ug·kg^−1^)	Clothianidin (ug·kg^−1^)	Imidacloprid (ug·kg^−1^)	Acetamiprid (ug·kg^−1^)	Thiacloprid (ug·kg^−1^)
1	0.64	<0	<0	0.21	1.23	1.95	0.65
2	0.53	<0	<0	0.15	1.4	1.97	0.53
3	0.47	<0	<0	0.57	1.21	2.07	0.58
4	0.40	<0	<0	0.15	1.22	1.95	0.50
5	0.48	<0	<0	0.67	1.26	1.95	0.64
6	0.52	<0	<0	0.32	1.21	1.93	0.51
7	0.61	<0	<0	0.15	1.33	1.94	0.50
8	0.42	<0	<0	<0	1.23	1.94	0.51
9	0.60	<0	<0	<0	1.22	1.95	0.51
10	0.55	<0	<0	0.46	1.26	1.97	0.51
11	0.73	<0	<0	0.21	1.43	2.00	0.54
12	1.03	<0	<0	1.39	1.33	1.99	0.52
13	0.96	<0	<0	<0	1.24	3.64	0.54
14	0.51	<0	<0	0.28	1.27	1.98	0.52
15	0.58	<0	<0	0.23	1.25	1.94	0.52
16	1.12	<0	<0	0.17	1.30	1.95	0.53
17	0.87	<0	<0	0.12	1.22	1.95	0.51
18	0.71	<0	<0	1.49	1.24	1.96	0.54
19	0.53	<0	<0	0.23	1.22	1.96	0.52
20	0.60	<0	<0	0.36	1.25	1.99	0.55
21	0.43	<0	<0	0.13	1.24	1.95	0.56
22	0.98	<0	0.07	1.58	1.46	2.06	0.52
23	0.88	<0	<0	<0	1.24	2.16	0.51
24	1.18	<0	<0	0.36	1.33	1.98	0.58
25	1.02	<0	<0	0.57	1.48	1.99	0.65
26	1.35	<0	<0	0.50	1.33	1.98	0.56
27	0.95	<0	<0	<0	1.27	1.96	0.54
28	1.56	<0	<0	0.37	1.46	2.06	0.53
29	0.64	<0	<0	0.64	1.23	1.93	0.51
30	0.89	<0	<0	0.11	1.25	3.39	0.52
31	0.55	<0	<0	0.68	1.44	1.95	0.51
32	0.81	<0	1	0.81	1.78	2.01	0.53
33	0.48	<0	<0	0.51	1.24	2.46	0.51

## Data Availability

The datasets generated during and/or analysed during the current study are available from the corresponding author on reasonable request.

## References

[1] Matsuda K., Buckingham S. D., Kleier D., Rauh J. J., Grauso M., Sattelle D. B. (2001). Neonicotinoids: insecticides acting on insect nicotinic acetylcholine receptors. *Trends in Pharmacological Sciences*.

[2] Jeschke P., Nauen R. (2008). Neonicotinoids-from zero to hero in insecticide chemistry. *Pest Management Science*.

[3] Wood T. J., Goulson D. (2017). The environmental risks of neonicotinoid pesticides: a review of the evidence post 2013. *Environmental Science and Pollution Research*.

[4] Craddock H. A., Huang D., Turner P. C., Quirós-Alcalá L., Payne-Sturges D. C. (2019). Trends in neonicotinoid pesticide residues in food and water in the United States, 1999-2015. *Environmental Health*.

[5] Fairbrother A., Purdy J., Anderson T., Fell R. (2014). Risks of neonicotinoid insecticides to honeybees. *Environmental Toxicology and Chemistry*.

[6] European Commission (2013). Commission implementing regulation (eu) no 485/2013 of 24 may 2013. amending implementing regulation (eu) no 540/2011, as regards the conditions of approval of the active substances clothianidin, thiamethoxam and imidacloprid, and prohibiting the use and sale of seeds treated with plant protection products containing those active substances. *Official Journal of the European Union*.

[7] Thrasyvoulou A., Tananaki C., Goras G. (2018). Legislation of honey criteria and standards. *Journal of Apicultural Research*.

[8] Yang C., Ran L., Xu M., Ren D., Yi L. (2019). In situ ionic liquid dispersive liquid–liquid microextraction combined with ultra-high performance liquid chromatography for determination of neonicotinoid insecticides in honey samples. *Journal of Separation Science*.

[9] Hou J., Xie W., Hong D. (2019). Simultaneous determination of ten neonicotinoid insecticides and two metabolites in honey and Royal-jelly by solid-phase extraction and liquid chromatography− tandem mass spectrometry. *Food Chemistry*.

[10] Si W., Wang S., Bai B. (2022). Zeolite H-beta as a dispersive solid-phase extraction sorbent for the determination of eight neonicotinoid insecticides using ultra-high-performance liquid chromatography—tandem mass spectrometry. *Applied Sciences*.

[11] Codling G., Al Naggar Y., Giesy J. P., Robertson A. J. (2016). Concentrations of neonicotinoid insecticides in honey, pollen and honey bees (Apis mellifera L.) in central Saskatchewan, Canada. *Chemosphere*.

[12] Mrzlikar M., Heath D., Heath E., Markelj J., Kandolf Borovsak A., Prosen H. (2019). Investigation of neonicotinoid pesticides in Slovenian honey by LC-MS/MS. *LWT--Food Science and Technology*.

[13] Proietto Galeano M., Scordino M., Sabatino L. (2013). UHPLC/MS-MS analysis of six neonicotinoids in honey by modified QuEChERS: method development, validation, and uncertainty measurement. *International Journal of Food Science*.

[14] Jovanov P., Guzsvány V., Franko M. (2013). Multi-residue method for determination of selected neonicotinoid insecticides in honey using optimized dispersive liquid–liquid microextraction combined with liquid chromatography-tandem mass spectrometry. *Talanta*.

[15] Jovanov P., Guzsvány V., Lazić S. (2015). Development of HPLC-DAD method for determination of neonicotinoids in honey. *Journal of Food Composition and Analysis*.

[16] Valverde S., Ares A. M., Bernal J. L., Nozal M. J., Bernal J. (2022). Determination of neonicotinoid insecticides in bee products by using ultra-high-performance liquid chromatography–tandem mass spectrometry. *Pesticide Toxicology*.

[17] Kavanagh S., Henry M., Stout J. C., White B. (2021). Neonicotinoid residues in honey from urban and rural environments. *Environmental Science and Pollution Research*.

[18] Tanner G., Czerwenka C. (2011). LC-MS/MS analysis of neonicotinoid insecticides in honey: methodology and residue findings in Austrian honeys. *Journal of Agricultural and Food Chemistry*.

[19] Ligor M., Bukowska M., Ratiu I. A., Gadzała-Kopciuch R., Buszewski B. (2020). Determination of neonicotinoids in honey samples originated from Poland and other world countries. *Molecules*.

[20] Woodcock B. A., Ridding L., Freeman S. N. (2018). Neonicotinoid residues in UK honey despite European Union moratorium. *PLoS One*.

[21] Chauzat M. P., Carpentier P., Martel A. C. (2009). Influence of pesticide residues on honey bee (Hymenoptera: apidae) colony health in France. *Environmental Entomology*.

[22] Han M., Wang Y., Yang Z. (2022). Neonicotinoids residues in the honey circulating in Chinese market and health risk on honey bees and human. *Environmental Pollution*.

[23] Mullin C. A., Frazier M., Frazier J. L. (2010). High levels of miticides and agrochemicals in North American apiaries: implications for honey bee health. *PLoS One*.

[24] Kasiotis K. M., Anagnostopoulos C., Anastasiadou P., Machera K. (2014). Pesticide residues in honeybees, honey and bee pollen by LC–MS/MS screening: reported death incidents in honeybees. *Science of the Total Environment*.

[25] Ozdemir N., Muz M. N. (2022). Neonicotinoid analysis in sunflower (helianthus annuus) honey of tekirdag province in turkey. https://www.researchsquare.com/article/rs-1683983/v2.

